# Reversal of an immunity associated plant cell death program by the growth regulator auxin

**DOI:** 10.1186/1756-0500-1-126

**Published:** 2008-12-02

**Authors:** Suresh Gopalan

**Affiliations:** 1 Present address : Department of Molecular Biology, Massachusetts General Hospital & Department of Genetics, Harvard University, Boston, MA 02114, USA; 2 DOE-Plant Research Laboratory, Michigan State University, East Lansing, MI 48824, USA; 3 Department of Plant Pathology, University of Kentucky, Lexington, KY 40546, USA

## Abstract

**Background:**

One form of plant immunity against pathogens involves a rapid host programmed cell death at the site of infection accompanied by the activation of local and systemic resistance to pathogens, termed the hypersensitive response (HR). In this work it was tested (i) if the plant growth regulator auxin can inhibit the cell death elicited by a purified proteinaceous HR elicitor, (ii) how far down the process this inhibition can be achieved, and (iii) if the inhibition affects reporters of immune response. The effect of constitutive modulation of endogenous auxin levels in transgenic plants on this cell death program was also evaluated.

**Results:**

The HR programmed cell death initiated by a bacterial type III secretion system dependent proteinaceous elicitor harpin (from *Erwinia amylovora*) can be reversed till very late in the process by the plant growth regulator auxin. Early inhibition or late reversal of this cell death program does not affect marker genes correlated with local and systemic resistance. Transgenic plants constitutively modulated in endogenous levels of auxin are not affected in ability or timing of cell death initiated by harpin.

**Conclusion:**

These data indicate that the cell death program initiated by harpin can be reversed till late in the process without effect on markers strongly correlated with local and systemic immunity. The constitutive modulation of endogenous auxin does not affect equivalent signaling processes affecting cell death or buffers these signals. The concept and its further study has utility in choosing better strategies for treating mammalian and agricultural diseases.

## Background

A well studied form of immunity against pathogens in the plant kingdom involves a rapid programmed cell death at the site of infection by the pathogen, associated with restriction of multiplication and spread of the pathogen, termed the hypersensitive response (HR) [1]. Often this HR cell death is accompanied by induction of broad spectrum resistance in uninfected parts of the plants, which is referred to as systemic acquired resistance (SAR). This process is conceptually similar to the requirement of immune activation accompanying apoptosis in some cell types [2]. Apoptosis and many other forms of programmed cell death and many of the components have been identified and extensively studied in different kingdoms [3]. The cell death program and its constituent components in plants is less well understood than the corresponding phenomena (e.g., of aptoptosis) in mammals and other organisms, though existence of mechanistic parallels has been demonstrated [4,5]. The point until which the cell death program can be reversed or inhibited has not been specifically addressed in any of these systems, which is one of the aspects addressed in this work.

The response of the plant host to a pathogen is intricately dependent on the physiological and developmental status of the plant, that are in turn controlled by signaling by different growth hormones and environmental conditions. Many gram-negative bacterial pathogens of plant, animal and human hosts e.g., *Pseudomonas*, *Erwinia*, *Xanthomonas*, *Ralstonia, Yersinia*, *Shigella *and *Salmonella *species encode a secretion system, termed the type III secretion system (TTSS) that enable them to secrete effector proteins that affect the host, many of which are directly translocated into host cells [6]. In the case of plants, the recognition of several bacterial effectors have evolved through a class of often intracellular LRR containing receptors and/or kinases, termed resistance (R) genes that recognize specific effectors [1]. The specific genetic or biochemical recognition of the bacterial component by the plant host triggers the rapid HR cell death program.

Harpins, unlike many other type III effectors were identified as proteins that are secreted by the bacteria into the media via the TTSS and by their ability to effect host cell death when purified protein is injected into the host apoplastic space (intercellular space) of leaves [7,8]. Despite this main difference many lines of evidence support the conclusion that harpin elicited cell death is programmed and shares many aspects of resistance associated cell death phenomena in plants [9-11]. While the receptor that recognize harpins is yet to be reported, a ca. 25 kDa protein is recognized in the membrane enriched fraction of *Nicotiana tabacum *by an anti-idiotypic antiserum to harpin from *Erwinia amylovora *(harpin_Ea_) (S. Gopalan and SY. He, unpublished results).

## Results and discussion

### Early inhibition and late reversal of harpin mediated cell death program by the growth regulator auxin

**Figure 1:**
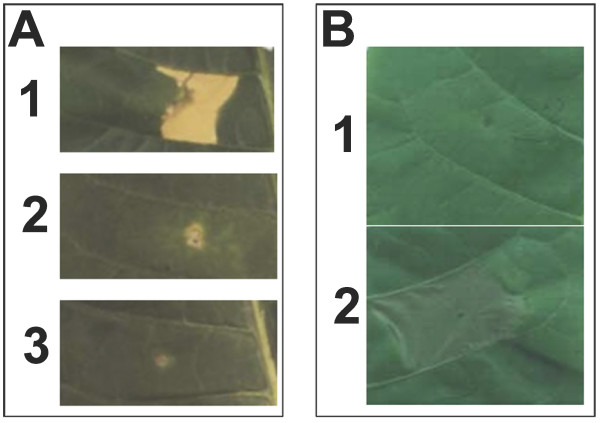
**Early inhibition and late reversal of harpin initiated cell death program by growth regulator auxin**. (A) Inhibition of HR cell death by auxin. Harpin (panel 1), Harpin in 50 μM 2,4-D (panel 2), or 50 μM 2,4-D (panel 3) was infiltrated into the apoplastic space of leaves of *Nicotiana tabacum *CV. Samsun NN, and symptom development was recorded 36 h later. (B) HR cell death program can be reversed till late in the process. Auxin (50 μM 2,4-D) – (panel 1) or buffer (panel 2) was infiltrated in the same area where harpin was infiltrated 6 hours earlier. The difference in appearance of the symptoms between Fig. 1(A) and 1(B) is due to the differences in the way the tissue dries after second infiltration of buffer. Harpin was used at 1 – 2 μM and purified as described earlier [10]. Symptoms were recorded 24 h after the first infiltration.

**Figure 2:**
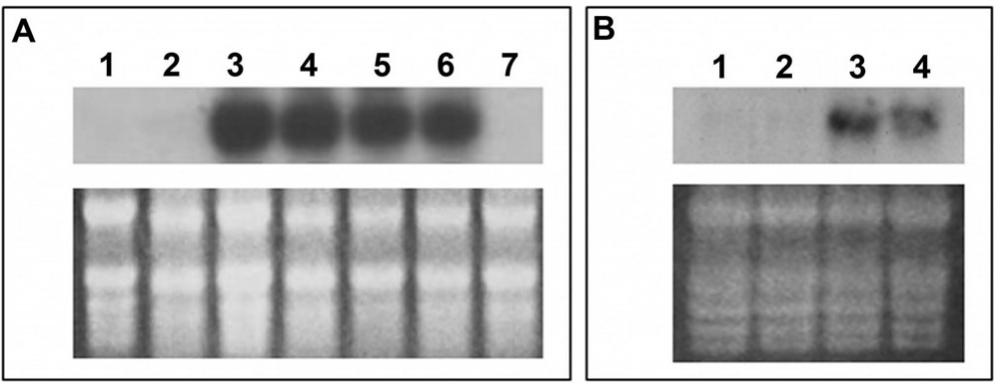
**Induction of defense genes are not affected by effect of auxin on harpin initiated cell death**. (A) Expression of *HIN1 *mRNA. Northern blot using RNA from leaves infiltrated with lane 1: Buffer 4.5 h; lane 2: 50 μM 2,4-D 4.5 h; lane 3: Harpin 4.5 h; lane 4: Harpin + 50 μM 2,4-D 4.5 h; lane 5: Harpin + 50 μM 2,4-D 24 h; lane 6: harpin – necrotic (9 h); lane 7: 50 μM 2,4-D 24 h. (B) Expression of *PR1 *mRNA. Lane 1: Buffer 24 h; lane 2: 2,4-D 24 h; lane 3: harpin 24 h; lane 4: Harpin + 2,4-D 24 h. 10 μg total RNA per lane from infiltrated area (A) or 1 cm region surrounding the infiltrated region (B) were used for the analysis. For panel B, leaf tissue was collected from region surrounding the infiltrated region because *PR1 *was not induced in the infiltrated area at these time points. RNA extraction, sequence and preparation of probes and subsequent procedures used have been described earlier [10]. rRNA bands of the RNA gel used for the Northern blot, visualized by staining with ethidium bromide, is shown in the lower frame of each panel.

Based on the well established concept that organismal homeostasis and key processes are controlled by the delicate balance between antagonistic death and survival signals [12], the hypotheses that the HR cell death program induced by a purified bacterial elicitor – harpin, can be reversed by a key plant growth regulator, auxin was tested [13,14]. To examine the effect of auxin on HR cell death, 50 μM auxin (2,4-D) and purified harpin_Ea _were coinfiltrated into the apoplast of *Nicotiana tabacum *cv. Samsun NN leaves. Whereas treatment of harpin in buffer elicited cell death – Fig. 1A-1, auxin completely inhibited harpin elicited cell death Fig. 1A-2. Inhibition could also be observed by co-infiltrating indole acetic acid (IAA) at 50 μM and harpin (data not shown).

To examine how farther down the process this inhibition can be achieved, auxin or buffer was infiltrated 6 h post-infiltration of harpin, in the same area where harpin was initially infiltrated. Under the conditions of the experiments, harpin elicited visible tissue flaccidity about 8 h post-infiltration (pi). Strikingly, the area infiltrated with auxin 6 h after initial infiltration did not show any cell death symptoms, whereas the buffer infiltrated area showed visible tissue flaccidity 3 h later and eventually showed HR symptom (Fig. 1B). These data indicate that the cellular commitment to die and the cell death program can be reversed by appropriate signals till late in the process.

### Cell death associated immune responses are operational despite early inhibition or late reversal of the program

As mentioned above the HR cell death is associated with local and systemic resistance in plants. The induction of marker genes strongly correlated with both these processes were tested. Induction of both the genes tested, *HIN1 *– a gene induced locally at the site of HR cell death [10], and *PR1 *– an established marker gene whose induction is tightly correlated with SAR, were not affected by inhibition of HR cell death by auxin – Fig. 2. While the precise contribution of HR cell death to resistance is not known, there are examples of cell death elicitation (spontaneous and induced) that can lead to resistance [15,16]. In contrast, there are also examples of or constitutive resistance without cell death [17,18]. An example of separate of HR cell death and resistance programs is the R gene (Rx) mediated recognition of potato virus X (PVX) [19]. Collectively, these data indicate that resistance phenomena while tightly associated with certain cell death processes, is triggered through a divergent signaling process that is strongly modulated by signals from cell death programs.

### Possible cellular programs effecting auxin based inhibition and reversal of cell death program and their relationship to pathophysiology

While the mode of action of the reversal of this cell death program need to be worked out, some clues suggested by signaling and physiological effects of auxin are, (i) involvement of ubiquitin mediated proteolysis, as signaling through many short-lived transcription factors is a hallmark of auxin response and the fact that TIR1, a ubiquitin ligase, is an auxin receptor mediating these responses [20,21], (ii) auxin causes extracellular acidification and cell wall loosening as opposed to alkalinization and crosslinking of cell wall during HR. Recent studies have revealed a role for auxin signaling during susceptible plant-pathogen interactions. In one case an miRNA induced by a resistance inducing pathogen associated molecular pattern (PAMP) – the peptide flagellin – has been shown to supress auxin signaling, and stimulating this arm of auxin signaling may promote susceptibility [22]. In another case, an increase in endogenous auxin levels and increase in symptom development by application of exogenous auxin during susceptible interactions have been observed [23]. One possible mechanistic implication in terms of pathophysiology of infection is that some aspect(s) of auxin signaling suppress cell death program and enhances disease susceptibility. This raises the intriguing possibility that the cell death during later stages of susceptible interaction of pathogen (typically considered necrosis) contributes to feedback resistance to some extent or possibly share some components with resistance associated cell death program like in HR, or both.

### How does auxin affect signaling modules to impair the cell death program?

In contrast to inhibition and reversal of the harpin elicited cell death program by exogenous auxin, transgenic plants constitutively modulated in their endogenous levels of free auxin (either increased 7 fold or decreased 10 fold), [24,25] were neither affected in their ability to elicit harpin or the bacteria *Pseudomonas syringae *pv. *syringae *61 (Pss61) induced cell death or the timing induction of death symptoms (Table 1). Similarly, exogenous auxin could not prevent cell death mediated by bacterial pathogens tested (*Erwinia amylovora *and Pss61 – Table 1 and data not shown). Earlier, it had been shown that auxin produced by some bacteria, can inhibit cell death elicited by an incompatible bacterial pathogen when coinfiltrated [26]. These data suggest the following testable hypotheses, (i) signals reprogrammed by constitutive modulation of endogenous auxin levels is not sufficient or equivalent to exogenous application of auxin in inhibition and reversal of cell death program, (ii) alternatively, the signaling modules are buffered by constitutive modulation of endogenous auxin levels (thus not able to express signals contributing to inhibition of the cell death program), and (iii) the difference in the inhibition of bacterially elicited HR cell death by auxin and by purified elicitor possibly reflects the extended and continuous delivery of cell death elicitors or auxin by bacteria, as opposed to single delivery of the elicitor and auxin in this study. Thus, having a purified elicitor of cell death facilitated this line of investigation.

**Table 1:** Constitutive endogenous modulation of auxin levels does not affect cell death

	**Relative free** **IAA level**	**Harpin**	**Pss61** **0.1 OD**	**Pss61** **0.05 OD**	**Pss61** **0.01 OD**
**Vector control**	1×	+	+	+	-

**IaaM #1**	~7×	+	+	+	+

**IaaL #3**	~0.1×	+	+	+	+

Transgenic plants used were *Nicotiana tabacum *cv. Samsun NN expressing (i) the tryptophan monooxygenase gene (*iaaM*) from *Agrobacterium tumefaciens*, (ii) the IAA-lysine synthetase (*iaaL*) gene from *Pseudomonas syringae *subsp. *savastanoi *that inactivates free auxin by conjugating it to lysine, from a constitutive 35S promoter, or (iii) the control vector integrated plants [24,25]. Relative IAA levels are as reported in the original publications. The leaves of these plants were infiltrated with harpin or *Pseudomonas syringae *pv. *syringae *(Pss61) at OD600 indicated. Development of cell death symptom was monitored at different time points and recorded as present (+) or no cell death (-) at 36 h post infiltration. There were no differences in the timing of appearance of cell death symptoms in all cases indicated with a +.

## Conclusion

In summary, these data demonstrate that a form of programmed cell death program in plants can be reversed till very late in the process and can be separated from the induction of associated disease resistance processes as evidenced by induction of strongly correlated transcriptional markers. The inhibition by auxin is akin to inhibition of apoptosis induced by growth or survival factors [12]. The observations presented here and the predicted mode of signaling pathways involved and their interplay have broader applications to modulate disease processes in different kingdom. Relevant examples include the recent demonstration that immune activation in adjacent cells (through TLR4) by signals from the dying cells is important for effective apoptotic therapy of certain tumors [2], and the reduction of toxicity associated with radiotherapy by using a TLR5 agonist through stimulation of antiapoptotic pathway [27]. Extensive studies of the molecular mechanisms of programmed cell death have identified many antagonistic signals, control points and gateways [28] and associated immune processes, but the point until which the cell death program can be reversed has not been specifically addressed. For example, the activation of the executioner caspases during apoptosis, unless inhibited rapidly by an inhibitor such as XIAP or degraded, is considered a point of no return in the cell death program [29].

The data presented here highlight the concept that a cell death program can be reversed till very late in the process and provides a framework to decipher the mechanism. Such knowledge should aid modulation cellular programs that involve certain forms of programmed cell death, and dissociating beneficial programs concomitantly activated with the cell death program in certain mammalian and agricultural disease conditions by choosing targets that maximize the utilization of both the programs.

## Competing interests

The author declares that they have no competing interests.

## Authors' contributions

The author (SG) designed executed and interpreted the experiments and wrote the manuscript.
